# A Novel Method to Accelerate Orthodontic Tooth Movement Using Low-Intensity Direct Electrical Current in Patients Requiring en-Masse Retraction of the Upper Anterior Teeth: A Preliminary Clinical Report

**DOI:** 10.7759/cureus.39438

**Published:** 2023-05-24

**Authors:** Rashad I. Shaadouh, Mohammad Y Hajeer, Rabab Al-Sabbagh, Mohammad Khursheed Alam, Ghiath Mahmoud, Ghassan Idris

**Affiliations:** 1 Department of Orthodontics, Faculty of Dentistry, Damascus University, Damascus, SYR; 2 Department of Orthodontics, Faculty of Dentistry, Hama University, Hama, SYR; 3 Department of Preventive Dentistry, College of Dentistry, Jouf University, Sakakah, SAU; 4 Department of Orthodontics, School of Medicine and Dentistry, University of Griffith, Griffith, AUS

**Keywords:** upper anterior teeth retraction, acceleration of orthodontic tooth movement, orthodontic tooth movement, retraction rate, en-masse retraction, electrical stimulation

## Abstract

Background: Shortening the duration of orthodontic treatment by speeding up the rate of tooth movement has become an essential goal for both orthodontists and patients. This preliminary report aimed to investigate the safety and effectiveness of a new intraoral removable electrical device in accelerating the en-masse retraction of the upper anterior teeth using low-intensity direct electrical current.

Methods: This prospective preliminary interventional clinical study was conducted at the Department of Orthodontics, Faculty of Dentistry, Damascus University, Syria, between March 2019 and February 2020. The sample consisted of six patients (four females and two males; mean age: 19.55 ± 0.89 years) whose initial diagnosis was class II division I malocclusion, and their treatment plan suggested the extraction of upper first premolars followed by en-masse retraction. The electrical stimulation was applied on the maxillary anterior region during the en-masse retraction phase using a specially fabricated removable device that was designed by two coauthors of this manuscript (RIS, MYH). Patients were asked to wear their own electrical devices inside their mouths for five hours daily. The primary outcomes were the en-masse retraction rate and duration. The secondary outcomes were safety and patient acceptance.

Results: The average total retraction rate during the treatment period was 0.97±0.06 mm/month. The total amount of retraction achieved during follow-up was 5.65 ± 0.85 mm, which was about 91.86% of the space resulting from the extraction of the upper first premolars. The mean treatment duration to complete the en-masse retraction was 5.66±0.81 months. No side effects of the electrical stimulation were found during the follow-up.

Conclusions: Low-intensity direct electrical current could be an effective method to accelerate orthodontic movement. The electrical accelerating device used in this study effectively increased the en-masse retraction rate of the upper anterior teeth without any side effects and with high patient acceptance.

## Introduction

Comprehensive orthodontic treatment with fixed orthodontic appliances takes a relatively long period, which in some cases may reach two or three years, especially if there is a need for teeth extraction [1]. This long-term treatment is considered one of the most important challenges facing both orthodontists and patients alike, and it may be the reason for patients' refusal of treatment [2]. In addition, the long duration of orthodontic treatment may be associated with many adverse effects, such as white spots, caries, gingival recession, and root resorption, as well as a lack of patient cooperation and treatment acceptance [3,4]. Therefore, shortening the duration of orthodontic treatment by speeding up the rate of tooth movement has become an essential goal for both the orthodontists and the patient, especially adults who want to finish their treatment in the shortest possible time for aesthetic and social concerns [5].

Many attempts have been made clinically and in vivo to find different methods that achieve faster results and reduce the duration of orthodontic treatment, including surgical methods such as traditional corticotomy [6], flapless corticotomy [7], laser-assisted flapless corticotomy [8], piezocision [9] and corticision [10] that depend mainly on inducing the regional acceleratory phenomenon (RAP). Or non-surgical methods such as low-level laser therapy (LLLT)[11], biomechanical appliance modification [12], and pulsed electromagnetic fields [13]. Those non-invasive techniques to accelerate orthodontic tooth movement have achieved promising results as surgical methods with greater patient acceptance [14].

On the other hand, the one-step retraction technique is another way that was described to reduce the treatment time after premolars extraction [15]. Several studies that evaluated the en-masse retraction of the upper anterior teeth following first premolars extraction have shown that it is a better technique compared to the two-step retraction method for several reasons, such as the shorter treatment time [1,2]. However, it is noteworthy to know that the mean en-masse retraction time has been estimated to lie between 9.94 and 12.60 months [2], which could also be reduced by different acceleration methods. 

After understanding the piezoelectric nature of bone, many studies have been conducted to evaluate the morphogenesis and remodeling of bone and surrounding tissues by direct electrical stimulation [16]. An animal study on three groups of cats was conducted by Davidovitch et al. to evaluate the effect of applying 15-20 microamperes of direct electrical current on periodontal tissues reported that electrical stimulation accelerates bone remodeling by increasing cellular enzymatic phosphorylation activities [16]. They also found that with a combination of electrical stimulation and mechanical force, teeth moved much faster than those treated with orthodontic forces alone [17]. Also, another animal study concluded that micro-electrical stimulation may play a role in the effective remodeling of the alveolar bone, thus moving the teeth more rapidly [18].

Kim et al., in a controlled clinical study on seven female patients, tested the effectiveness of low-intensity electric current in accelerating the retraction of the upper canines. They found that the electric current stimulated the tooth movement and made it faster by 30% [19].

Finding an appropriate source of electrical stimulation inside the mouth cavity was the main problem with applying this method in clinical practice. Kim et al. used a small electrical device mounted on the upper canine bracket to speed up the movement of the canines [19]. However, this device was suitable only for canine retraction and could not be applied to other cases, such as en-mass retraction or anterior teeth decrowding. From this point, a new intraoral electrical device was designed by the research team at the Orthodontic Department at Damascus University, Damascus, Syria, in collaboration with an electronic engineer. The proposed system can be a simple and easy-to-use device that can provide the appropriate electric current to stimulate the orthodontic tooth movement during the en-masse retraction of the upper anterior teeth. This preliminary report aimed to investigate the safety and effectiveness of this new removable device in accelerating the en-masse retraction of the upper anterior teeth.

## Materials and methods

Study design and settings

This trial was a pilot interventional clinical study evaluating the effectiveness of a new device for accelerating orthodontic tooth movement. It was conducted at the Department of Orthodontics, Faculty of Dentistry, Damascus University, Syria. The Research Ethics Committee of the Damascus University Faculty of Dentistry approved this trial protocol (DN-040423-39). The trial’s protocol was not registered since it was a pilot study.

Patients recruitment

Six patients whose initial diagnosis was class II division I malocclusion were randomly selected from the patients registered in the diagnostic records of the Department of Orthodontics, Faculty of Dentistry at Damascus University, between March 2019 and April 2019. The inclusion criteria were adult patients aged between 17-25 years who have skeletal and dental class II division I relationship, and their treatment plan suggested extraction of the upper first premolars. The exclusion criteria were: (i) anterior deep bite, (ii) severe overjet (more than 10 mm), (iii) moderate or severe dental crowding, (iv) loss of teeth on the upper arch, (v) previous orthodontic treatment, (vi) patients with health conditions or long-term medication, (vii) periodontal breakdown, and (viii) bad oral hygiene. After explaining the protocol of this trial to patients, none of them refused to participate, and written informed consent was obtained from all of them. 

Treatment sequence

Extraction of First Premolars 

The maxillary first premolars were extracted at the beginning of the treatment before the adhesive of the orthodontic brackets.

Anchorage Reinforcement

Self-drilling orthodontic mini-screws (1.6 × 8 mm; 3S screw, HUBIT Orthodontics, Gyeonggi, South Korea) were used as a skeletal anchorage. It was inserted bilaterally between the upper second premolar and the first molar roots.

Leveling and Alignment

Votion^TM^ MBT prescription and pre-adjusted fixed orthodontic appliances (Ortho Technology, West Columbia, South Carolina, United States) were used in this trial. The archwires (Ortho Technology) were changed every three weeks in this phase as the following sequences: 0.014-inch nickel-titanium (NiTi), 0.016 × 0.016-inch NiTi, 0.017 × 0.025-inch NiTi, 0.019 × 0.025-inch NiTi, and 0.019 × 0.025-inch stainless steel (SS). After three weeks of reaching the final 0.019 × 0.025-inch SS, crimpable hooks were added distal to the upper canines.

En-masse Retraction of the Upper Anterior Teeth

After completing the leveling and alignment phase, the en-masse retraction was started by applying 250 g of force on each side using NiTi closed coil springs (NT3® closed coil, American Orthodontics, Sheboygan, Wisconsin, United States) stretched from the crimpable hook to the mini-screw. An intraoral force gauge (040-711-00; Dentaurum GmbH & Co. KG, Ispringen, Germany) was used to evaluate the retraction force every two weeks.

Low-Intensity Direct Electrical Current Application

The electrical stimulation was applied on the maxillary anterior region during the en-masse retraction phase using a specially fabricated removable device that was designed by two of the authors (RIS, MYH). Patients were asked to wear their electrical devices inside their mouths for five hours daily.

The electric accelerating device

The electric accelerating device was invented by two researchers (RIS and MYH) in the Orthodontic Department at Damascus University, who designed the external structure of the device that carries the electrical circuit and other parts in collaboration with an electronic engineer (AMZ), who designed the electrical circuit. The main goal was to reach a small device that is easy to use inside the mouth by the patient himself and capable of applying a low-intensity direct electric current with specific parameters to the periodontal tissues in the area of the upper anterior teeth to benefit from the ability of electric current to accelerate bone metabolism in accelerating orthodontic tooth movement.

**Figure 1:**
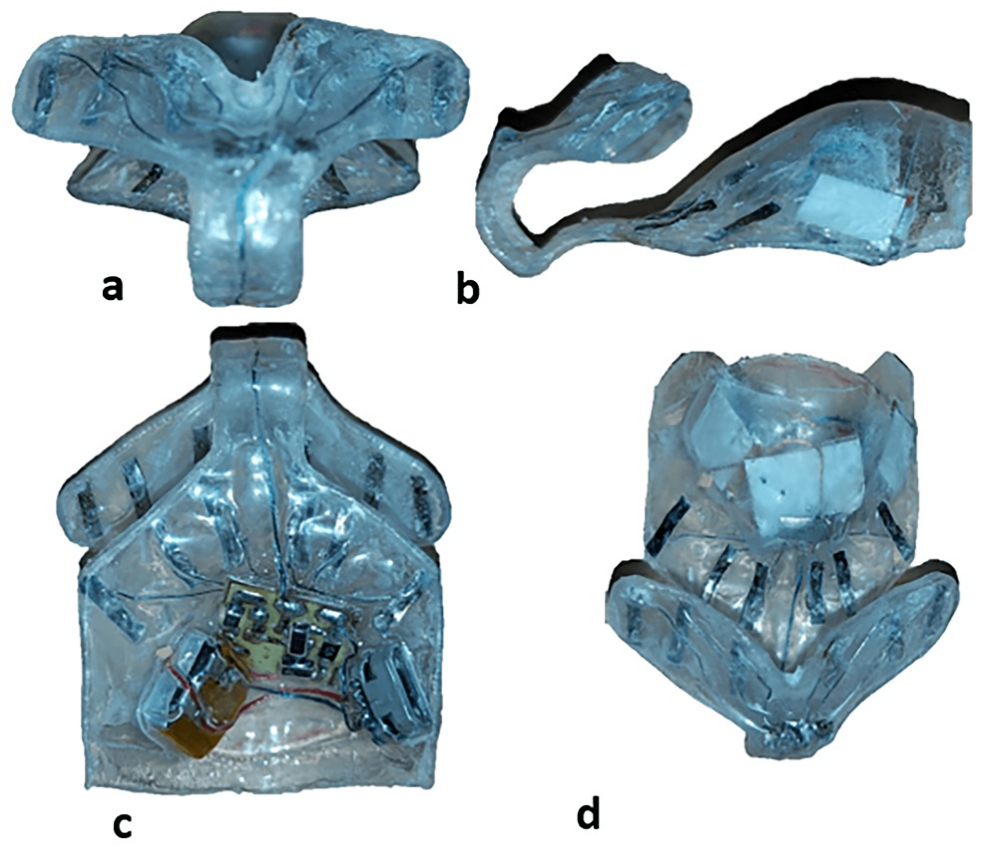
The electrical accelerating device. (a) Frontal view of the device; (b) Side view of the device; (c) Occlusal view of the device; (d) Top view of the device.

**Figure 2:**
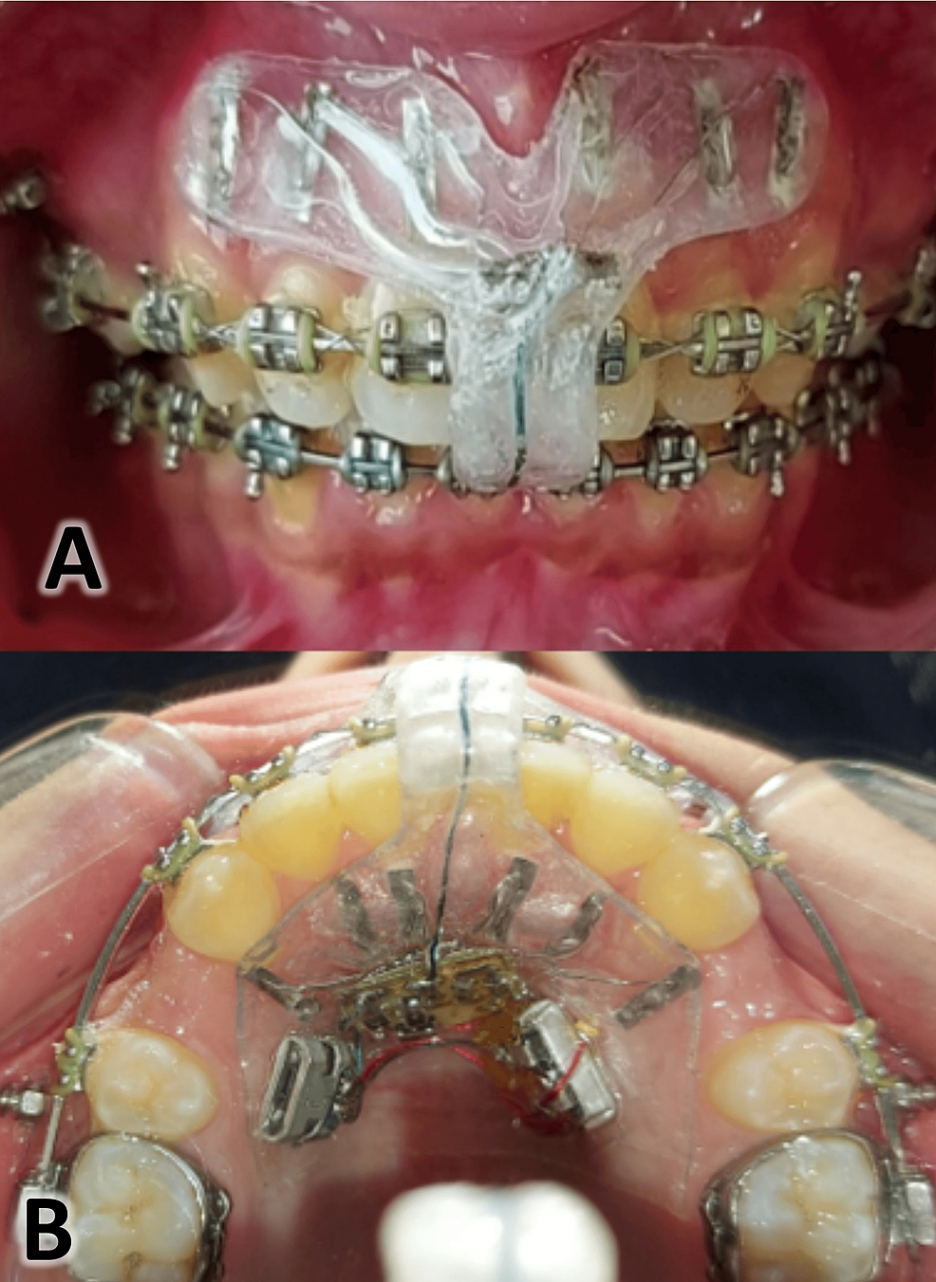
The electrical accelerating device. (A) Frontal view of the device inside the patient's mouth; (B) Occlusal view of the device inside the patient's mouth.

The device was designed to be fitted with the anterior region of the maxilla in a way that allows the electric current to pass across the upper anterior teeth area (buccally-palatally; Figure 1 and Figure 2).

The device was formed of two flexible vacuum-formed plates (3A MEDES®, Goyang, Korea) of 1 mm thickness, fitted to each other, that enclose the internal components of the device. The device consisted of two parts connected: buccal and palatal, which were tightly attached to the patient's gums. The palatal part covered the anterior region of the palate, palatal to the upper anterior teeth, and fitted tightly to the gingival tissues. It extended from the distal of the upper canine to the distal of the canine on the opposite side and extended from the free gingiva to the distal of the second premolars sagittally. It contained (i) a small electrical circuit for supplying a specific intensity electric current (15-20 µA for each tooth), (ii) a small battery (LP401314 Li-polymer battery, 3.7 volts, 50 mA; LiPol Battery Co., Ltd, Shenzhen, China), (iii) battery charging port (USB micro-B type; Elecbee, Dongguan, Guangdong, China), (iv) electrical wires that carry electric current to the electrodes, (v) electrodes (the positive electrode) that were placed on the opposite surface of the gum, in direct contact with it, and distributed over the six front teeth, its dimensions 2 × 6 mm, and (vi) connecting wires between all the parts.

**Figure 3:**
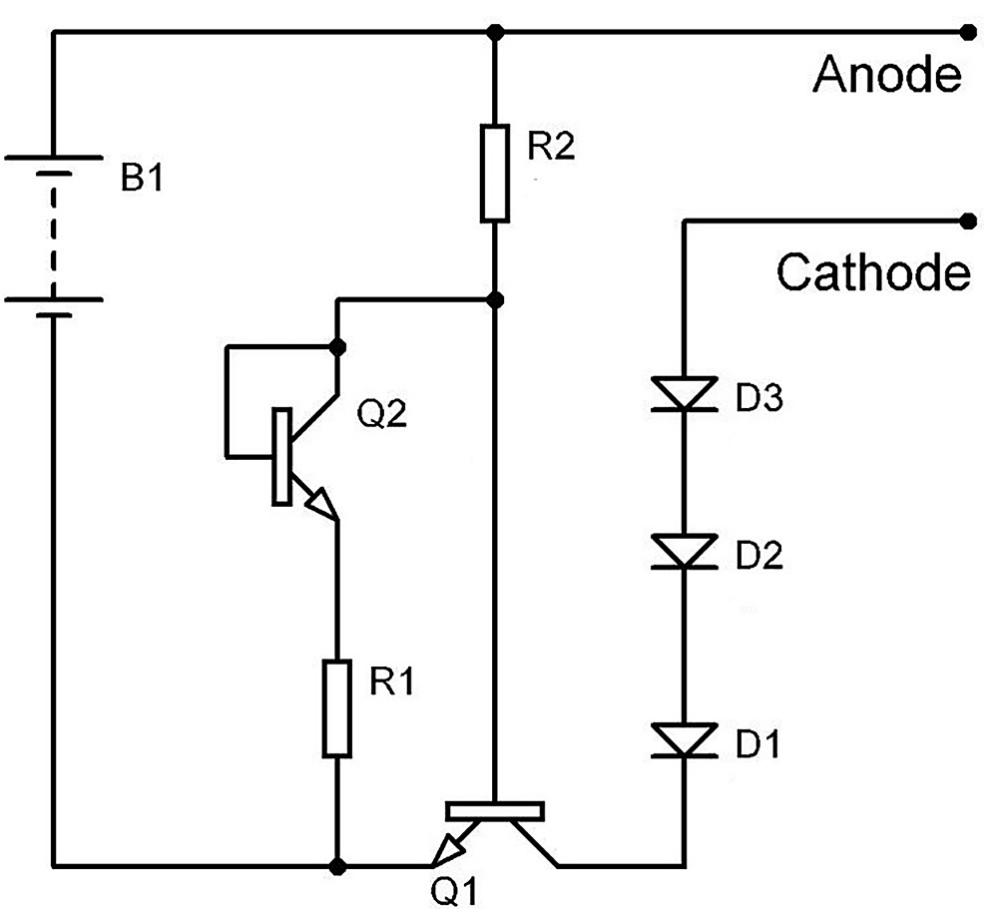
Schematic diagram of the electrical circuit. B1: Battery, Q1,Q2: Transistors; R1,R2: Resistors; D1, D2, D3: Diodes.

The buccal part covered the attached gingiva of the anterior teeth and extended from the middle of the upper canine to the middle of the canine on the opposite side with attention to the frenulum of the upper lip, vertically extended from the mucogingival junction to the free gingiva. It was tightly attached to the patient's gums. This part contained electrical wires that carry electric current to the electrodes and electrodes (the negative electrode) that were placed on the opposite surface of the gum, in direct contact with it, and distributed over the six front teeth, its dimensions 2 × 6 mm. The electric circuit, which had a 1 cm^2^ size, was made up of a collection of transistors and resistors and was designed to provide a direct electric current of 15-20 A for each tooth of 1.5 volts (Figure 3).

The application of the device inside the mouth by the patient led to the closing of the circuit and the passage of electric current with the required intensity (15-20 µA for each tooth) between the electrodes, passing through the soft tissues and the periodontal ligament. The passage of current from the buccal side of the teeth to the palatal side was according to the direction of the movement of the anterior teeth during the en-mass retraction, so it was supposed to enhance the dental movement and bone metabolism around the teeth and thus accelerated the orthodontic movement.

Outcome measures 

Primary Outcomes Measure: Rate and Duration of the En-masse Retraction

The monthly rate of the upper anterior teeth movement was assessed on study models. The retraction rate was calculated by dividing the amount of retraction (millimeters) by the duration (month) to estimate the retraction rate. Model casts from alginate impressions were made at the following times: T0: at the end of the leveling and alignment stage, T1: one month after the en-masse retraction began, T2: after two months, T3: after three months, T4: after four months, T5: after five months of the en-masse retraction began, and TF: at the end of the en-masse retraction (when reaching a class I canine’s relationship and a correct overjet). The distance between the incisor edge of the central incisors and the medial end of the third palatal fold was measured on the digital photographs of the model casts using the ImageJ program‏ (National Institutes of Health, Bethesda, Maryland, United States) as the way described by Khlef et al. [7] to detect the amount of incisors retraction. The monthly retraction rate was calculated by dividing the retraction distance by the time passed. 

Secondary Outcome Measures: Safety and Patient Acceptance 

Patients were examined at each appointment for any adverse reaction that may have occurred because of electrical stimulation. They were also asked to report any problem they may face. Patients were asked at the end of the observation about their willingness to undergo the treatment again, recommending this procedure to a friend, and if getting used to the electrical device was easy, medium, or difficult.

Reliability assessment of the used method

The measurements on the dental casts were performed again two weeks after the first evaluation. The interclass correlation coefficients (ICCs) were used to determine random errors, and paired sample t-tests were used to detect systematic errors.

## Results

Baseline sample characteristics

From the 64 patients registered in the diagnostic records of the Orthodontic Department who met the inclusion criteria, six patients were randomly selected and enrolled in this pilot study after obtaining their consent (four females, two males; mean age: 19.55 ± 0.89 years). The data of these six patients were analyzed at the end of the follow-up period with no withdrawals. Table 1 shows the characteristics of the patients before treatment began.

**Table 1:** Baseline characteristics of the sample at the beginning of the treatment SD: standard deviation

Number of patients	6
Gender (male/female)	2/4
Age (years) ± SD	19.55 ± 0.89
Crowding (no/mild)	4/2
Facial height (normal/ excessive)	1/5
Overbite (normal/shallow)	1/5
Posterior crossbite (no/yes)	6/0
Pre-retraction extraction space	6.15 ± 0.39

The reliability of the used method

The calculated ICCs indicated high reliability for the performed measurements, as its result ranged from 0.978 to 1. On the other hand, insignificant differences were found between the two sets of measurements (P>0.05) when paired sample t-tests were applied. Therefore, minor and unimportant systematic errors were discovered.

Rate of en-masse retraction

The mean rate of the anterior teeth movement was 0.96±0.08 mm/month in the first month of observation (Table 2); then it increased in the second and third months to become 1.02±0.05 and 1.00±0.13 mm/month, respectively. In the last two months of follow-up (i.e., the fourth and fifth months), the en-masse retraction rate decreased slightly to 0.99±0.08 and 0.92± 0.10 mm/month, respectively. The average total retraction rate during the treatment period was 0.97±0.06 mm/month. The total retraction achieved during follow-up was 5.65 ± 0.85 mm, which was about 91.86% of the space resulting from the extraction of the upper first premolars. The mean treatment duration to complete the en-masse retraction was 5.66±0.81 months.

**Table 2:** Descriptive statistics of the en-masse retraction rate (mm/month) SD: standard deviation, N: number of patients, Max: maximum value, Min: minimum value

	Intervals	N	Mean ± SD	Median	Max value	Min value
Upper anterior teeth retraction rate	T0-T1 (month 1)	6	0.96 ± 0.08	0.95	1.10	0.84
	T1-T2 (month 2)	6	1.02 ± 0.05	1.02	1.08	0.94
	T2-T3 (month 3)	6	1.00 ± 0.13	1.00	1.17	0.81
	T3-T4 (month 4)	6	0.99 ± 0.08	0.99	1.10	0.88
	T4-T5 (month 5)	6	0.92 ± 0.10	0.91	1.08	0.80
	T0-TF	6	0.99 ± 0.05	1.007	1.04	0.90

The safety of the intervention and patient acceptance

No side effects of the electrical stimulation were found during the follow-up, such as irritation, gingival ulceration, burning, discomfort, swelling, or bleeding. In addition, none of the patients reported any side effects while using the device between the follow-up appointments. All patients in this trial reported that they would recommend this technique to their friend as a good and easy way to speed up the orthodontic treatment and that they had no problem with repeating this treatment. Four of six patients stated that it was easy to get used to the accelerating device, and the other two patients found it moderately difficult. 

## Discussion

The purpose of this preliminary clinical study was to evaluate the efficiency of low-intensity electrical stimulation in accelerating orthodontic teeth movement using a new manually fabricated electrical device that was designed for this goal. The maxillary first premolars were extracted at the beginning of the treatment before starting the leveling and alignment phase to avoid the effect of extraction on the tooth movement rate by stimulating the RAP [20] and also to ensure that the socket in the extraction area healed and did not affect the tooth movement [21].

The en-masse retraction technique was studied in this trial as a recent systematic review showed the superiority of one-step retraction over traditional two-step retraction techniques regarding treatment duration, anchorage loss, skeletal and dental changes, and aesthetic results [2]. Mini-implants were used to provide absolute skeletal anchorage, and 8-10 mm height hooks were used to apply horizontal forces parallel to the archwire so that the force axis passed from or was close to the center of resistance of the anterior teeth to achieve as much bodily movement as possible [4]. The application protocol of electrical current in this study was chosen based on the results of Kim et al.'s study, which found that low-intensity electric stimulation of 15 μA for five hours per day was effective in accelerating the retraction of the upper canines by about 30% [19]. This study was the first clinical study that evaluated the effect of low-intensity direct electric current on the rate of orthodontic movement during en-masse retraction of the upper anterior teeth. Therefore, the results of this study were compared with other studies that evaluated other methods in accelerating en-masse retraction.

In this study, the en-masse retraction of the upper anterior teeth took an average period of 5.66±0.81 months, and the average total en-masse restoration rate during the treatment course was 0.99±0.05 mm/month. To evaluate the effectiveness of low-intensity electric current, the results of this study were firstly compared with those of other studies that used mini-implants supported en-mass retraction without any acceleration method. Upadhyay et al., in three studies published in 2008 and 2009, found that the mean time to complete en-masse retraction was 8.61±2.2 months [22], 9.2 months [23], and 9.4 months [24]. Al-Sibaie et al. reported a mean en-masse retraction duration of 12.90 months [1]. Finally, Tunçer et al. found that the en-masse retraction time in the control group was 9.27±2.55 months [25]. As a result, the en-masse retraction time was lower by three to six months when it was combined with low-intensity electric stimulation. However, it should be noted that there were some differences between these studies and the current trial in terms of the intensity of the forces used, as it was 150 g in most of them [1,22-24], while the retraction forces in this trial were 250 g on each side. Or in terms of the base archwire used for the retraction, where it was 0.016 × 0.022-inch [25], 0.017 × 0.025-inch [22-24], or 0.019 × 0.025-inch [1].

On the other hand, compared with the results of studies that evaluated different accelerating methods with mass retraction, it was observed that both flapless corticotomy and traditional corticotomy achieved greater acceleration compared to the current study. According to Khlef et al., the average retraction time was 4.04±1.10 and 3.75±2.14 for flapless corticotomy and traditional corticotomy, respectively [4]. At the same time, the retraction rate with these methods was 1.26 and 1.38, respectively [7]. It is noteworthy to know that these studies were very similar to the current study in terms of included participants and en-masse retraction protocol. Compared to the low-level laser therapy, Lalnunpuii et al. found that the average retraction rate of the upper anterior teeth was 0.68 × 0.23 mm/month in the experimental group [26]. Also, the mean rate of en-masse retraction in Samara et al.'s study using photobiomodulation therapy to accelerate teeth movement was 1.08 × 0.54 mm per month [27], which was almost equal to the results of this study.

Based on these data, it can be said that the low-intensity electric current effectively accelerated the en-masse retraction of the upper anterior teeth. However, further controlled studies should be conducted to confirm these results. No important side effects were noted by the clinician or reported by patients during the treatment course; this may indicate the safety and reliability of the used electrical device. And could prove the ability to use it inside the mouth. However, more controlled studies with more participants are needed to confirm these findings. Recent research has focused on patient-reported outcome measures during accelerated orthodontics [28], which should also be covered in future research work.

The results of the current study showed that 100% of patients answered yes to the questions of recommending this treatment to a friend and undergoing this treatment procedure again if needed. This high percentage indicated a very high acceptance by patients. This could be because the pain and discomfort associated with this procedure were less and tolerable and did not affect their daily social activities. On the other hand, only two patients found it moderately difficult to adapt to the electrical accelerating device, while the rest of them found it easy to adapt to it. This result agrees with the previous findings and confirms the high acceptability of the applied procedure during en-masse retraction [29,30].

Limitations

Although the current study is the first to evaluate low-intensity direct electrical current in the context of accelerating en-masse retraction of the six maxillary anterior teeth, it has some shortcomings. The absence of a control group was the main limitation of this study, in addition to the small number of patients included. Pain, discomfort, and functional impairment associated with the use of this technique were not systematically evaluated in this study. Finally, long-term complications such as dental vitality should be considered in future studies.

Generalizability

The results of this study may not be generalizable because it was conducted on a small number of patients with specific malocclusions, and strict inclusion criteria were followed. Therefore, more clinical studies should be conducted with different types of malocclusions to obtain better generalizability.

## Conclusions

Based on the results of this study, low-intensity direct electrical current could be an effective method to accelerate orthodontic movement. The electrical accelerating device used in this study effectively accelerated the en-masse retraction of the upper anterior teeth without any side effects. The acceptance of the device by patients was very high. The protocol mentioned in this study for electrical stimulation appeared to be a practical procedure that could shorten the orthodontic treatment time.
